# From Emollients to Biologicals: Targeting Atopic Dermatitis

**DOI:** 10.3390/ijms221910381

**Published:** 2021-09-26

**Authors:** Lorenzo Salvati, Lorenzo Cosmi, Francesco Annunziato

**Affiliations:** Department of Experimental and Clinical Medicine, University of Florence, 50134 Florence, Italy; lorenzo.salvati@unifi.it (L.S.); francesco.annunziato@unifi.it (F.A.)

**Keywords:** atopic dermatitis, therapy, skin, type 2 inflammation, emollients, barrier repair therapy, dupilumab, nemolizumab, JAK-inhibitors

## Abstract

Atopic dermatitis (AD) is the most common chronic inflammatory skin disease and significantly impacts patients’ lives, particularly in its severe forms. AD clinical presentation varies over the course of the disease, throughout different age groups, and across ethnicities. AD is characterized by a spectrum of clinical phenotypes as well as endotypes. Starting from the current description of AD pathogenesis, this review explores the rationale of approved AD therapies from emollients to biologicals and introduces novel promising drugs.

## 1. Introduction

“It is true the longest drought will end in rain.”—Robert Frost (from On Looking Up By Chance At The Constellations, 1928).

Atopic dermatitis (AD) treatment is nowadays moving towards a real paradigm shift, especially for patients affected by severe AD. In fact, the advent of biological therapies is changing the management and course of severe AD, giving patients a treatment option that is safer than traditional systemic therapies and with excellent clinical results. Notwithstanding emollients are still the mainstay of treatment, the systemic therapeutic approach to AD has changed in recent years moving from traditional systemic therapies to monoclonal antibodies and more recently introducing small molecules. However, none of the advances that we are witnessing today would have been possible without the study of the pathogenic mechanisms that underlie this inflammatory skin disease.

## 2. Atopic Dermatitis: Molecular and Clinical Features

AD is a chronic inflammatory cutaneous disease with prevalence rates up to 20% in children and 10% in adults [1,2,3]. Multifactorial pathogenesis, characterized by a complex interplay of epidermal barrier dysfunction, host genetics, environmental factors, and immune perturbations, underlies AD [1,4].

### 2.1. Genetic Factors

Genetic factors include loss-of-function (LoF) mutations in the gene encoding filaggrin (FLG) [5]. Filaggrin is a major structural protein, whose monomers aggregate and align keratin bundles with an important role in ensuring the mechanical strength and integrity of the stratum corneum, and whose metabolites contribute to forming the natural moisturizing factor [6]. Between 10% and 40% of patients with atopic dermatitis present with LoF mutations of the FLG gene and have a disease phenotype characterized by early-onset, higher severity, face and hands involvement, palmar hyperlinearity, increased susceptibility to Staphylococcal infections, asthma and food allergy [7].

### 2.2. Type 2 Response and Beyond

Historically, experimental models which are based on the atopy patch test showed a biphasic pathogenetic process in AD: in the acute phase, AD is a T helper (Th)2 cell-mediated disease, while a switch to Th1 cells promotes the chronic phase [8]. As a consequence of the epidermal barrier damage, antigens penetrate the skin, chemokines (CCL17, CCL22) are produced, and epithelial cytokines and alarmins (IL-25, IL-33 and TSLP) are released [9,10]. These events result in the activation of skin-resident innate lymphoid cells type 2 (ILC2) and polarize Th cells towards Th2 with the production of IL-4, IL-13 and IL-5 [11]. In addition to the activation of type 2 inflammation, pathways including Th1, Th17, and Th22 are also involved resulting in mixed inflammation [12,13,14]. Acute lesional AD skin shows mainly Th2 and Th22 immune responses. Persistence of Th2 and Th22 and sustained activation of Th1 and Th17 responses characterize chronic AD [15]. The chemokine CCL17, also known as thymus and activation-regulated chemokine (TARC), a chemoattractant of Th2 cells, correlates with AD clinical severity, at both the baseline and during therapy [16].

### 2.3. Atopic-Type: Endotypes and Phenotypes in Atopic Dermatitis

AD is a heterogeneous disease and includes a complex spectrum of endotypes according to the evaluation of molecular mechanisms in different groups of patients [17]. Differences are not only in relation to the stage of the disease, but the patient’s age and ethnicity also contribute [18,19]. In fact, considering the clinical phenotype, the polarization of immune response and the barrier defect, differences exist between pediatric compared to the adult population. In infants, AD is generally acute and mainly involves the face and the extensor surfaces of the limbs, but also the trunk, while in young children, particularly flexural folds are involved. In children, AD lesional skin lacks Th1 infiltrate differently from adults [20,21]. The shared signature of AD across ages is Th2/Th22-skewed, yet the differential expression of specific Th2/Th22-related genes portrays heterogenetic age-specific molecular pathways. [22]. African American patients with AD display a dominant Th22 response, but not Th17, while Asian patients with AD are characterized by lower Th1 response [20].

AD is clinically characterized by acute (oozing, oedema, and erythema) and chronic (xerosis, and lichenification) lesions that can coexist during flares. As a result of pruritus, excoriations are often present. AD is classically a clinical diagnosis that is mainly based on morphology and distribution (both spatial and temporal) of skin lesions, disease course, coexisting conditions, personal and family history of atopy [4,23]. Several clinical phenotypes of AD have been described and are defined morphologically (nummular dermatitis, prurigo nodularis-like lesions, erythroderma, lichenified dermatitis, follicular/papular dermatitis and pompholyx [dyshidrosis]) and topographically (flexural or periorificial or occurring on the face, lips, eyelid, head and neck, hand and foot, or nipple) [23]. The atopic type is extremely complex. Immune cells involved in the inflammatory process and genetic alterations define endotypes that reflect disease presentation.

## 3. Therapeutic Approach to Atopic Dermatitis

**Figure 1:**
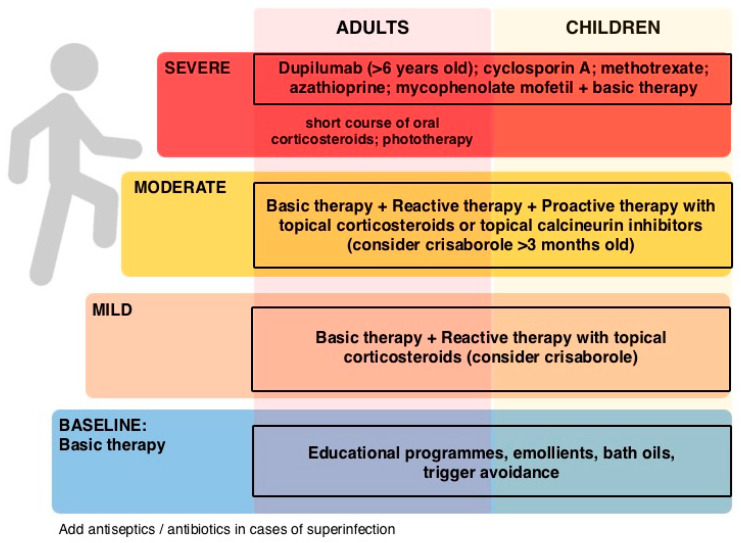
Atopic dermatitis therapeutic stepwise approach adapted from [24,25,26].

To date, therapeutic management depends on the patient’s age and disease severity (Figure 1). Management requires a multi-pronged approach aimed at controlling inflammation (anti-inflammatory), itching (anti-pruritic), bacterial superinfection (anti-bacterial) and skin barrier restoration (moisturizer). The treatment options for AD in adults and children are defined in practice clinical guidelines and summarized in Figure 1 [24,25,26,27]. The approach is stepwise in consideration of disease severity as assessed on the basis of the clinical features, extent and location of skin lesions, pruritus intensity, and sleep disturbance [26]. These characteristics can be measured with scores such as the SCORAD (SCOring Atopic Dermatitis) which classifies severe AD when it is above 50 [28]. The most common symptom in all patients with AD is itching. Already in the 1970s, before the pathogenesis was known in detail, it was written on Rook that the fundamental principle of AD therapy is to prevent scratching [29]. In the 1990s, Caputo and Ackerman wrote that “the single most important step in the management of atopic dermatitis is prevention of pruritus, without itching and scratching, there can be no atopic dermatitis” [30]. Today, we know that pruritus signaling pathways may be histamine-dependent or rely on other molecules such as IL-31 and are mediated by C and Aδ fibers [1]. Pruritus in AD is only in part mediated by histamine [31,32,33,34]. Oral histamine H1 receptor antagonists are routinely used to try to relieve itching in AD patients, but there is currently no high-level evidence to support or refute that nonsedating antihistamines as monotherapy reduce pruritus in patients with AD [35,36,37]. When oral antihistamines are used in combination with other therapies, a better control of pruritus was observed compared to placebo [38,39]. Oral histamine H4 receptor antagonist (adriforant) showed clinical efficacy on inflammatory skin lesions in a phase 2 study but with a reduction in pruritus similar to placebo [40].

### 3.1. Emollients

The basic therapy, which may be sufficient in mild forms, is based on emollients whose action is aimed at restoring the integrity of the skin barrier, thus counteracting xerosis and reducing itching. The barrier damage in AD is determined by various factors (genetic and environmental) which in many ways contribute to enhance the protease activity at the level of the stratum corneum [41,42]. It becomes clear the reason why, as barrier repair therapy, emollients are the mainstay of AD treatment [43]. Regular daily use of the emollient extends the interval between relapses and attenuates the intensity of acute phases with steroid-sparing effect. The use of emollients from birth might contribute to the prevention of AD in children, as shown in prospective studies [44,45]. Emollients should be used liberally and frequently, at least 250 g per week in adults [24]. Composition of the product is important: emollients with fewer ingredients, fragrance-free and without known allergenic preservatives such as parabens, and preferably composed of physiological lipids (such as ceramides), should be preferred [46].

### 3.2. Topical Immunesuppressants

For topical therapy, anti-inflammatory drugs such as topical corticosteroids are used, particularly in acute phases [46,47,48]. Among the most powerful topical steroids, there is clobetasol propionate, but the most used in clinical practice are those of class III, generally used in single evening application (beclomethasone dipropionate, betamethasone dipropionate, betamethasone valerate, budesonide, desoximetasone, diflucortolone valerate, fluticasone propionate, methylprednisolone aceponate, mometasone furoate) [24,46,49]. The proactive therapy (e.g., twice a week application) is useful in maintaining disease control over time and reducing relapses [24,50]. Topical calcineurin inhibitors (pimecrolimus cream 1% and tacrolimus ointment 0.03% and 0.1%) are also important anti-inflammatory drugs especially to be used in more sensitive skin areas (face, intertriginous sites, anogenital area) [24,49]. Proactive therapy with twice-weekly application of tacrolimus ointment may reduce relapses [51,52]. Effective sun protection is generally recommended [53].

### 3.3. Topical Antibiotics

Skin microbiome has a central role in topical therapy [54]. Up to 90% of AD patients present with *Staphylococcus aureus* skin colonization [55]. Bacterial communities antagonize each other and change over the course of the disease. Notably, skin flare-ups are species-specific and characterized by increased *Staphylococcus aureus* colonization in disease flares [56]. While topical therapy with fusidic acid or mupirocin is indicated in bacterial superinfection, Gram-negative bacterial lysates such as Vitreoscilla filiformis have been shown to improve AD reducing local inflammation [57,58]. It should also be noted that in patients with AD there is a higher prevalence of *Staphylococcus aureus* resistance to fusidic acid compared with healthy controls [59,60]. Moreover, *Staphylococcus aureus* isolates from children with AD differ in antimicrobial resistance profiles from those in non-atopic nasally colonized children [59,61].

### 3.4. Traditional Systemic Therapy

Traditional systemic therapy includes corticosteroids, cyclosporine A and other immunosuppressants [62]. Corticosteroids should be used for short-term treatment (up to 1 week) in the acute phase, preferentially in adults with severe AD [25]. The daily dose of systemic corticosteroids should be adjusted to and not exceed 0.5 mg/kg/day. Long-term use is not recommended [25]. Many randomized clinical trials (RCTs) indicate the efficacy of cyclosporine A versus placebo in AD [63]. Cyclosporine A can be used at 3–5 mg/kg/die up to 2 years [25,64]. Azathioprine (2–3 mg/kg/die), methotrexate (5–15 mg/week) and mycophenolate mofetil (2 g/day) can also be used in the management of severe AD [65,66,67,68,69].

### 3.5. Monoclonal Antibodies

The currently available treatments of AD are summarized in Table 1.

In addition to emollients, traditional topical and systemic therapy, the armamentarium against AD is nowadays enriched by biological therapy approved in severe forms [70]. Monoclonal antibodies can directly target a cytokine or a receptor with the aim of modulating the inflammatory response [71,72].

Dupilumab is a fully human monoclonal antibody that binds to the IL-4Rα chain [73]. This chain is found in the IL-4 receptor where it dimerizes with the γ chain and in one of the two isoforms of the IL-13 receptor where it dimerizes with IL-13Rα1 to form the IL-4/IL-13 receptor [74,75]. Consequently, dupilumab acts by blocking both IL-4 and IL-13 pathways and inhibiting the Th2 response which is central to the pathogenesis of AD. Dupilumab causes potent inhibition of Th2-associated chemokines (CCL17, CCL18, CCL22, and CCL26), decreases mRNA expression of hyperplasia-related genes (K16 and MKI67), and inhibits IL-17/IL-22-modulated genes (CXCL1, CXCL2, PI3, IL-23p19/IL-23A, and S100 genes) [76]. Dupilumab was the first monoclonal antibody approved for the treatment of moderate-to-severe AD in adults (in 2017), in adolescents aged 12 to 18 years (in 2019) and in children aged 6 to 11 years (in 2020) whose eczema is not adequately controlled by topical therapies, or when those therapies are not advisable [77,78,79,80,81,82,83]. Dupilumab should be combined with daily emollients and may be combined with topical corticosteroids as needed [25,84]. Data from the extension of phase 3 study (NCT01949311) to 76 weeks of treatment in adults demonstrated that efficacy was maintained over time and in terms of safety the most frequent adverse reaction was conjunctivitis in 10.7% of cases, followed by injection site reactions [85]. It is important to note, however, that in real life, as shown by the Italian DADReL study group, the incidence of conjunctivitis in AD patients treated with dupilumab tends to be higher than RCTs, approximately 40% [86]. A recent meta-analysis has confirmed that conjunctivitis is the most common adverse event, reported in 26.1% of patients [87]. The pathogenesis of these side effects is at present unknown [88,89,90]. In children, the treatment was well tolerated in the long term and the most frequent adverse reaction was nasopharyngitis [91]. Nonetheless, dupilumab has revolutionized the treatment of severe AD with significant improvement of symptoms and outcomes of patients, as confirmed in a recent systematic review including 1845 subjects >12 years treated 16 to 52 weeks, and yet is the only biological therapy approved in AD [92]. In the absence of well-powered head-to-head trials comparing all possible combinations of systemic immunomodulatory treatments, a systematic review and network meta-analysis demonstrated that dupilumab and cyclosporine may have better short-term effectiveness than methotrexate and azathioprine for the treatment of AD in adults [93]. Retrospective studies have shown longer drug survival for dupilumab compared to cyclosporine in severe AD [94,95].

Many further monoclonal antibodies are under study in AD, mainly targeting type 2 inflammation [96,97,98]. Tralokinumab and lebrikizumab are anti-IL-13 monoclonal antibodies that bind soluble IL-13 thus preventing IL-13Rα heterodimerization with IL-4Rα and consequent signaling via the IL-4R [96,97,98]. They both induced clinical improvement compared to placebo in AD patients: tralokinumab was superior to placebo at 16 weeks of treatment and it was well tolerated up to 52 weeks of treatment [99,100]. In a phase 2 study lebrikizumab was effective in reducing AD symptoms, even itching [101]. A phase 2a study of fezakinumab, anti-IL-22 monoclonal antibody, showed promising results compared to placebo, especially in patients with severe AD and particularly in those with high levels of IL-22 [102,103]. Tezepelumab, anti-TSLP monoclonal antibody, in combination with topical steroids did not show efficacy in adults with moderate to severe AD in a phase 2 study [104]. A proof-of-concept clinical trial of etokimab, anti-IL-33 monoclonal antibody showed rapid and sustained clinical benefit; a phase 2 study is under way (NCT03533751) [105].

Considering increased Th17 skewing in some AD endotypes, secukinumab, an anti-IL-17A monoclonal antibody, was investigated in AD patients, but there was no significant improvement compared to placebo [106]. IL-17C antagonist (MOR106) in experimental models reduced skin inflammation [107,108], but clinical studies (NCT03568071, NCT03864627, NCT03689829) were prematurely stopped for futility.

Different results are observed, blocking the OX40-OX40L axis using GBR 830, a humanized monoclonal antibody against OX40 which is a costimulatory receptor expressed by activated T cells [109,110]. This approach resulted in improved clinical outcomes in a phase 2a study, and determined significant progressive reductions in Th1, Th2, Th17/Th22 mRNA expression in lesional AD skin [109]. KHK4083, a fully human anti-OX40 monoclonal antibody, was effective and safe in a phase 1 study of Japanese patients with moderate-to-severe AD [111]. A phase 2 study has just been completed (NCT03703102).

Being pruritus crucial in AD pathogenesis, diminished quality of life, and poor sleep, therapies aiming to block the itch-scratch cycle have been investigated. Nemolizumab, an anti-IL-31Rα monoclonal antibody, was effective in controlling pruritus in AD patients [112,113,114]. A phase 3 study of 16 weeks duration demonstrated that in patients with moderate-to-severe AD the treatment with subcutaneous nemolizumab in addition to topical immunosuppressants resulted in a greater reduction in pruritus than placebo [115]. In patients treated with nemolizumab signs of eczema ameliorated, although that some patients reported worsening AD as an adverse event [115]. Studies evaluating the long-term efficacy and safety of nemolizumab in moderate-to-severe AD with pruritus are under way (NCT03989206).

### 3.6. Other Therapies and Upcoming Therapies

Crisaborole is a selective phosphodiesterase 4 (PDE4) inhibitor. In the topical 2% ointment formulation, it has been approved in 2016 for the treatment of mild to moderate AD in adults and pediatric patients from 2 years of age with ≤40% body surface area affected [116,117]. Crisaborole reduced inflammation-modulating Th2 and Th17/Th22 pathways in AD lesional skin and improved the barrier function reversing epidermal hyperplasia/proliferation [118].

A novel frontier in the treatment of AD is represented by JAK-inhibitors, small molecules that can be used both in the topical formulation thanks to their low molecular weight (about 300 kDa lower than the skin barrier threshold), and in the systemic oral formulation [119,120]. These include delgocitinib, ruxolitinib, and tofacitinib as topic formulations, baricitinib, upadacitinib, abrocitinib, and gusacitinib as systemic drugs [121,122]. Delgocitinib (pan-JAK-inhibitor) in ointment formulation improved clinical signs and symptoms in children and in adults with AD and it was well tolerated [123,124,125]. In 2020 delgocitinib ointment was approved in Japan and it was the first topical JAK-inhibitor to be available [126]. Ruxolitinib (JAK1/JAK2 inhibitor) cream provided rapid and sustained improvements in symptoms and reduced pruritus in adults with AD [127,128]. Additionally, tofacitinib (JAK1/JAK3 inihbitor with limited effect on JAK2) ointment displayed good results in a phase 2a study [129]. Baricitinib, an oral selective JAK1/JAK2 inhibitor, improved the clinical signs and symptoms of moderate-to-severe AD in phase 3 studies and was effective and safe in the long term [130,131,132,133].

Treatment with upadacitinib or abrocitinib, oral selective JAK-1 inhibitors, resulted in significant clinical benefit in moderate-to-severe AD patients and was well tolerated [134,135,136,137,138]. In addition, gusacitinib a dual JAK-SYK inhibitor showed strong efficacy with rapid onset of action and reduced systemic inflammation in moderate-to-severe AD [139,140]. 

## 4. Conclusions

**Figure 2:**
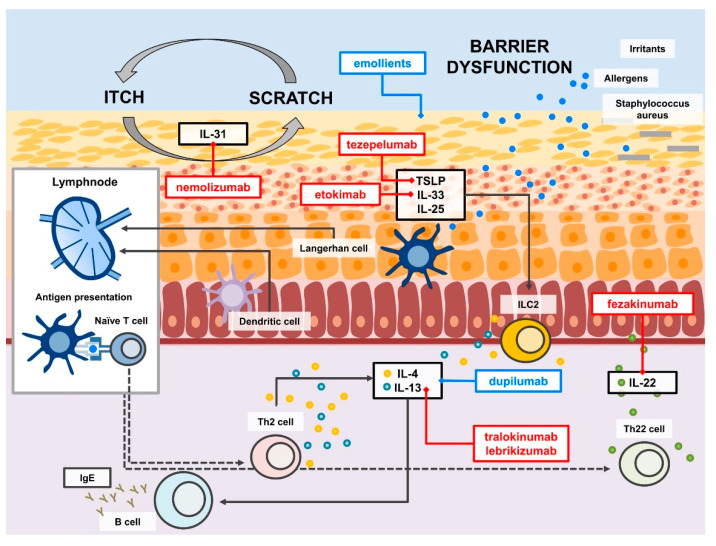
Main molecular pathways in atopic dermatitis targeted by emollients and biologicals. Approved available treatments are in blue boxes.

AD is a complex heterogeneous disease as shown by the broad range of disease phenotypes and endotypes; thus, the therapeutic approach must be personalized for the single patient and endotype-driven. Treatment personalization should be based on an integrated approach including characteristics of the patient and the disease. The main factors that should be considered are age, ethnicity, gender (e.g., pregnancy and lactation), morphology and localization of skin lesions, the severity of disease in terms of both body surface area involved and intensity of symptoms (especially pruritus), duration of disease, frequency of AD relapses, response to previous AD treatments, coexisting conditions (e.g., mucosal atopy, immunodeficiency, etc.), possible concomitant therapies, and impact on patient’s quality of life. The future prospect of personalized therapeutic options in AD will be the real-life use of validated AD-specific biomarkers both in the selection/stratification and monitoring of AD patients. Old and new treatments of AD target molecular mechanisms fundamental in disease pathogenesis (Figure 2). Over the last few years, biological therapies and JAK-inhibitors have rapidly and promisingly expanded the armamentarium against AD, so that the end of the drought does not seem so far.

## Figures and Tables

**Table 1:** Current available topical and systemic treatments in atopic dermatitis (as of September 2021).

Treatment	Recommendation
**Topical treatments**	
Emollients	Use daily
Topical corticosteroids	Short-term in the acute phase/proactive therapy
Topical calcineurin inhibitors	Skin sensitive areas/proactive therapy
Crisaborole	Mild-moderate AD
Topical JAK-inhibitors	Delgocitinib approved in Japan
**Systemic treatments**	
Corticosteroids	Short-term in severe AD
Cyclosporine A	Chronic severe AD
Azathioprine Mycophenolate mofetil	If cyclosporine A is not effective or not indicated
Methotrexate	Long-term maintenance
Dupilumab	Moderate-severe AD (>12 years) Severe AD (>6 years)
Oral JAK-inhibitors	Baricitinib in moderate-severe AD
